# The Role of Cognition in Divergent Thinking: Implications for Successful Aging

**DOI:** 10.3390/brainsci13101489

**Published:** 2023-10-21

**Authors:** Laura Colautti, Virginia Maria Borsa, Giulia Fusi, Maura Crepaldi, Massimiliano Palmiero, Francesca Garau, Natale Salvatore Bonfiglio, Jessica Giannì, Maria Luisa Rusconi, Maria Pietronilla Penna, Luca Rozzini, Alessandro Antonietti

**Affiliations:** 1 Department of Psychology, Catholic University of the Sacred Heart, 20123 Milan, Italy; alessandro.antonietti@unicatt.it; 2 Department of Human and Social Sciences, University of Bergamo, 24129 Bergamo, Italy; virginiamaria.borsa@unibg.it (V.M.B.); giulia.fusi@unibg.it (G.F.); maura.crepaldi@unibg.it (M.C.); jessica.gianni@unibg.it (J.G.); marialuisa.rusconi@unibg.it (M.L.R.); 3 Department of Clinical and Experimental Sciences, University of Brescia, 25136 Brescia, Italy; luca.rozzini@unibs.it; 4 Department of Communication Sciences, University of Teramo, 64100 Teramo, Italy; mpalmiero@unite.it; 5 Department of Pedagogy, Psychology, Philosophy, University of Cagliari, 09123 Cagliari, Italy; f.garau91@gmail.com (F.G.); penna@unica.it (M.P.P.); 6 IRCCS Centro San Giovanni di Dio Fatebenefratelli, 25125 Brescia, Italy; nbonfiglio@fatebenefratelli.eu

**Keywords:** creativity, divergent thinking, executive functions, crystallized intelligence, cognitive reserve, cognition, aging

## Abstract

Promoting active and successful aging has become crucial to improve quality of life in later adulthood and reduce the impact of cognitive decline. Increasing evidence suggested that the ability to think creatively (e.g., via divergent thinking), similar to cognitive reserve, could represent a beneficial factor against the negative effects of aging. However, there is still little evidence investigating the relationships between divergent thinking, cognitive functions, and cognitive reserve in late adulthood. The present study explored these relationships in a sample of 98 individuals ranging from 61 to 88 years old (mean age: 72.44 ± 6.35). Results showed that visual, but not verbal, divergent thinking was affected by aging. Interestingly, visual divergent thinking performance was predicted by both the cognitive component of crystallized intelligence and cognitive reserve. Only the crystallized component of intelligence was found to mediate the aging effect on visual divergent thinking performance. These results suggest that in later adulthood a potential shift strategy to prior knowledge and semantic components over executive and control components of cognition could underlie a preserved ability to think divergently and, plausibly, creatively. Limitations of the study and implications for successful aging are discussed.

## 1. Introduction

As life expectancy has increased, it has become crucial to sustain and promote active aging [1]. Several factors contribute to active aging, and, among them, cognitive functioning plays a pivotal role in supporting everyday life duties and preserving autonomy and personal growth [2,3]. However, there is evidence that some cognitive abilities are more susceptible than others to life changes and to the consequent physiological decline [4]. For instance, cognitive functions that pertain to the crystallized intelligence domain are mainly preserved, while cognitive mechanisms associated with fluid intelligence and supporting cognitive control—like executive functions (EFs)—usually are more susceptible to decline [1,4,5,6,7,8]. Cognitive decline is characterized by structural and functional changes at a neural level involving a widespread network of brain regions and, in particular, fronto-parietal networks [9,10,11,12]. It follows that older adults (OAs) may present impairments in those abilities that mainly underlie cognitive control, which is fundamental in everyday life [13].

In recent decades, increasing evidence reported that creative thinking, and in particular divergent thinking (DT), may be preserved in aging (for a systematic review, see [14]) and may support cognitive functioning, with beneficial effects on both healthy and clinical populations [3,15,16,17,18,19]. Thinking creatively concerns the ability to generate a product or an idea that is both (i) unexpected and unusual and (ii) useful and appropriate to the context [20], breaking automatic responses for developing alternative behaviors, especially in situations that are new to the individual, and coping with possible everyday life difficulties [16,17]. In this vein, the ability to think divergently, that is, the ability to find more than one solution to open-ended demands [21], is a complex construct that involves both crystallized components of intelligence (i.e., semantic and autobiographical memories) to draw on for the development of new ideas and fluid-processing components (i.e., EFs) aimed at facilitating semantic associations (which are pivotal in the generation of creative ideas), inhibiting automatic thinking, and changing the attentional focus flexibly [22]. Therefore, DT is primarily a cognitive measure useful to investigate the individual’s creative potential [23,24].

DT is sustained by wide-spread neural networks and in particular by (i) the default mode network (DMN), (ii) the salience network (SN), and (iii) the executive control network (ECN). The DMN, which encompasses predominantly the medial prefrontal cortex (MPC), medial temporal lobes, posterior cingulate cortex (PCC), precuneus, and inferior parietal lobule (IPL), is supposed to be fundamental for imaginative thinking, generation of ideas, and retrieval and association of autobiographical and semantic knowledge [25,26,27]. The SN, which involves predominantly the anterior cingulate (ACC) and insular cortices, is fundamental for monitoring functions [28,29] and switching between DMN and ECN functions (e.g., [28,29]). Finally, the ECN, encompassing mainly lateral prefrontal and anterior inferior parietal regions, is crucial for goal-directed cognition and executive functioning, such as working memory and prepotent response inhibition [25,26].

A well-supported claim in the literature is that the DMN-ECN coupling, also modulated by the SN, reflects the dynamic interplay between spontaneous and controlled modes of thought, with DMN activated for idea generation but regulated by ECN in order to maintain specific task goals [26,29,30,31]. Interestingly, according to the Default–Executive Coupling Hypothesis of Aging (DECHA hypothesis; [25,32]), the functional coupling between DMN and ECN could be altered in aging, leading older adults to rely less on declining control processes (subtended predominantly by the ECN), and thereby rely more on crystalized intellectual capacities (mainly subtended by the DMN), to support goal-directed behaviors [13]. This potential shift in strategies is reflected in DT performance. Indeed, DT does not decline steadily during aging and it is assumed that OAs count more on crystallized components of intelligence (semantics) rather than fluid ones (cognitive control) [33], adopting a compensation mechanism. In this way, individual differences recorded in creative tasks between older and younger adults may be due to other cognitive abilities functioning, such as working memory or speed processing [3,34,35].

DT has also been linked to cognitive reserve (CR), which underlies the ability to perform a task or achieve an objective by recruiting alternative strategies and cognitive processes when the usual strategies are no longer possible, as can happen in the aging process [36,37,38]. CR can be increased throughout the lifespan by everyday life experiences and whether the individual is exposed to proper environmental stimuli, being a resource against age-related cognitive decline [38,39]. The educational level, occupational status, and commitment to cognitively stimulate leisure activities are possible factors that contribute to enhancing CR and are usually considered CR proxies [39]. Evidence showed the presence of a relationship between CR and DT in late adulthood [36,37,40,41], indicating the possibility that enhancing creative thinking may increase CR, thereby supporting cognitive functioning.

Nevertheless, to date, there are a few studies concerning such an issue, and also considering the key role of components related to cognitive functioning. Thus, due to the properties of DT and its relationships with crystallized and fluid-processing components and CR, the investigation of the relationships among these constructs in aging appears to be important.

### Aims

According to the literature, the present study, firstly, aimed at investigating potential aging effects on DT abilities in both verbal and visual domains. Secondly, it explored the contribution of both crystallized and fluid (i.e., EFs) components of cognition in predicting performance in DT tasks. Finally, we investigated if crystalized components of intelligence mediate the relation between aging and DT, in line with the DECHA hypothesis, and therefore a potentially more “semanticized” over “controlled” cognitive processing in the elderly [13,42].

## 2. Materials and Methods

### 2.1. Participants

One hundred and thirty-three participants were recruited from two Italian regions, namely, Lombardy (70.6%) and Sardinia (28.6%).

All participants included in the sample had to meet the following inclusion criteria: age ≥ 60 years old; the absence of global cognitive impairments as defined by Mini-Mental State Examination (MMSE [43]) ≥ 24; psychological profile in the normal range; no history of neurologic impairments or neurosurgical interventions; and the absence of psychiatric disease or history of alcohol or drug addiction. From the initial sample of 133 participants, an additional screening procedure was carried out on the basis of neuropsychological tests administered during the experimental session (for further details, see Section 2.2. Procedure). The final sample consisted of 98 individuals (45 females; mean age = 72.44 years, SD = 6.35; mean education = 12.30 years, SD = 4.64).

### 2.2. Procedure

Data were collected from April 2021 to December 2022. All participants read and provided the informed consent. Two individual in-person sessions were scheduled, each of them lasting about 60 min, to evaluate DT, cognitive abilities, CR, and mood state. Participants who exhibited a pathological score in at least one neuropsychological test were excluded from the statistical analyses. All participants took part in the study on a voluntary basis. No incentive was provided.

The study was conducted according to the Declaration of Helsinki [44] and was approved by the Institutional Ethical Committee of the University of Bergamo and by the one of the Catholic University of the Sacred Heart in Milan.

### 2.3. Instruments

#### 2.3.1. Global Cognitive Functioning

Mini-Mental State Examination (MMSE [43]) is a screening test to evaluate global cognitive functioning. It is composed of 30 items investigating seven cognitive domains (temporal and spatial orientation, words registration, attention and calculation, words recall, language, and constructive praxis). A cut-off score of above 24 indicates a normal global cognitive functioning.

#### 2.3.2. Neuropsychological Testing

Trail Making Test (TMT [45]) is composed of two parts that measure, respectively, visual search mechanisms (part A) and divided attention mechanisms (part B). The scoring relies on the time (in seconds) taken to complete the two parts of the task. A measure of attentional shifting (TMT B-A) is obtained by subtracting the time related to part B from the time related to part A. Procedure of part A: from a sheet, participants need to link, using a pen, numbers from 1 to 25 with a continuous line. Procedure in part B: from another sheet, numbers and letters need to be linked by a continuous line, alternately and progressively (e.g., 1-A-2-B-3-C). 

Stroop Test, short version [46], is used to assess inhibitory self-control. It is composed of three parts, according to which participants are required to read the names of colors reported in black ink (first part), then to name the colors of some circles (color naming) (second part), and finally to name the ink color of written color names (third part). The stimuli of each part are reported vertically on different pages that the examiner turns quickly. An interference Stroop effect score (STROOP_I) is computed by subtracting the mean response times (in seconds) of the first and the second parts from the third part of the test according to the formula TI = T3 − (T1 + T2)/2.

Symbol Digit Modality Test, oral version (SDMT [47]), evaluates the speed of information processing. Participants have to associate the number corresponding to specific symbols, according to an association matrix provided, within 90 s. The score is computed by calculating the number of correct associations.

Digit Span Backward (Digit_bw [48]) is a measure of verbal working memory span. Participants have to listen to a sequence of numbers and then have to repeat it in the reverse order. The test ends when participants repeat all the sequences (up to the last pair of 9 numbers) or until they miss a consecutive pair of sequences of the same length. The score is computed considering that the longest sequence is correctly repeated.

Digit Span Forward test (Digit_fw [48]) is a measure of verbal short-term memory. Participants listen to a sequence of numbers and then have to repeat it in the same order. The test ends when participants repeat all the sequences (up to the last pair of 9 numbers) or until they miss a consecutive pair of sequences of the same length. The score is computed considering that the longest sequence is correctly repeated.

Story recall, 6 December [49], is used to investigate verbal episodic memory. Participants hear a short story regarding a fictitious event, read by the experimenter, and then are required to repeat all details that they remember, immediately after (immediate recall) and after a 10 min delay (delayed recall). Two different subscores are calculated considering a grid of words and details that participant provides during the immediate and delayed recall. A total score is provided by combining both the immediate and the delayed recall scores.

Wechsler Adult Intelligence Scale IV (WAIS IV), Vocabulary subtest (WAIS_voc [50]), is used to assess semantic components and crystallized intelligence. Participants are asked to provide the definition of some words, presented one after another, belonging to the Italian language. For each definition, a score is assigned (2 = the word is correctly defined; 1 = the definition is vague or not elaborated; 0 = the definition is incorrect or is absent) and a total score is calculated by adding the score obtained for each word. In total, there are 30 words. The test stops when the participant is unable to provide three consecutive word definitions or provides incorrect ones.

#### 2.3.3. Cognitive Reserve

Cognitive Reserve Index questionnaire (CRIq [51]) is proposed to investigate CR and consists of three parts, referring to the individual’s educational experiences, work experiences, and activities carried out during leisure time, i.e., CRIq school, CRIq work, and CRIq leisure activities, respectively. The type and frequency of activities engaged by participants related to each area are investigated, referring to the activities carried out from 18 years old up to the present. Activities engagement is investigated and divided based on weekly, monthly, and annual frequencies. Scoring for each of the areas is carried out. It is also possible to obtain a total score derived from the sum of the three parts.

#### 2.3.4. Mood State

Depression Anxiety Stress Scale (DASS [52]) is a 4-point Likert scale (ranging from 0 = It has never happened to me, to 3 = It happened to me most of the time) composed of 21 items investigating symptoms concerning three subdimensions: depression (Cronbach’s α = 0.82), anxiety (Cronbach’s α = 0.74), and stress (Cronbach’s α = 0.85). The subject must respond referring to the frequency with which each symptom occurred in the last week. A total score representing the general levels of distress (Cronbach’s α = 0.90) is provided as well, by summing all responses. 

#### 2.3.5. Divergent Thinking

Two tasks, from the TTCT [53], were selected to evaluate both verbal and visual DT.

The Alternative Uses Task (AUT) assesses verbal DT, by asking participants to find as many different and original uses of a cardboard box as possible, within 10 minutes. Responses for each item were scored according to the criteria described in the manual, considering three indexes: fluency (number of reliable answers provided), flexibility (number of semantic categories the answers belong to; it represents the variety of shifts in responses), and originality (rarity, innovation, and creativity of the responses according to the manual’s sample: 0 points = responses provided by ≥5% of 500 people; 1 point = responses provided by 2–4.99% of 500 people; and 2 points = responses provided by <2% of 500 people or responses not listed in the manual). 

The Line Test (LT) assesses figural DT. Participants are asked to draw as many figures as possible starting from two parallel lines (30 pairs of parallel lines were provided), within 10 min. For each item, responses were scored according to the manual and considering three indexes: fluency (number of reliable answers provided), flexibility (number of semantic categories the answers belong to; it represents the variety of shifts in responses), and originality (rarity, innovation, and creativity of the responses according to the manual’s sample: 0 points = responses provided by ≥5% of 500 people; 1 point = responses provided by 2–4.99% of 500 people; and 2 points = responses provided by <2% of 500 people or responses not listed in the manual). 

### 2.4. Statistical Analyses

The sample size for regression models was computed considering f^2^ as a measure of effect size. In detail, we assumed a medium effect size (f^2^ = 0.15), an α = 0.05, a power (1-β) of 0.80, number of total predictors = 8, and number of tested predictors = 3. Results yielded a sample size of 77 individuals. 

All individual scores were transformed into standardized z-scores for statistical analyses. Four participants were excluded from the analyses because of missing data or abnormal z-scores values (± 3 SD). Therefore, the final sample entered into statistical analyses included 94 participants. Standardized scores of both visual and verbal DT subscales (i.e., fluency, flexibility, and originality) were averaged in order to obtain a composite score, respectively, for visual and verbal creativity. In correlation analyses, both parametric (Pearson’s r) and nonparametric (Spearman’s rho) coefficients were computed in order to explore the relations between sociodemographic variables (i.e., age and education), DASS scores, cognitive tests scores, and DT scores. Then, partial correlations between cognitive outcomes, CRIq total, and DT scores were calculated, controlling for both age and education. An additional EF composite score (mean of STROOP_I and TMT B-A) was computed by combining the Stroop test’s interference scores with TMT shifting index (TMT B-A) and was used in hierarchical regression models.

Two separate hierarchical regression analyses were performed, considering both DT measures (i.e., visual and verbal) as dependent variables. In both regression analyses, age, education, and gender were entered as predictors in the first model, measures of EFs (i.e., executive composite score, Digit Span Backward, and SDMT) in the second model, measures of crystallized intelligence (WAIS_voc) in the third model, and cognitive reserve index (CRIq total) in the fourth model. Additional mediation models were then tested in order to investigate the effects of executive/fluid processing mechanisms (executive composite score), crystallized intelligence (WAIS_voc), and cognitive reserve index (CRIq_tot) as potential mediators of the relationship between age and visual DT.

## 3. Results

Table 1 and Table 2 reported the mean and SD of sociodemographic variables, neuropsychological tests, and creativity tests. 

### 3.1. Correlations

As expected, age and education were significantly correlated to almost all cognitive outcomes, except for verbal episodic memory (see Table 3). Significant correlations were found between age, education, and visual DT composite score (r = −0.406, *p* < 0.001; r = 0.220, *p* = 0.033). Notably, considering visual and verbal DT subscales, we found that age correlated with all visual subscales and only with verbal originality subscale (only a trend: *p* = 0.06) (see Table 4). 

Moreover, significant partial correlations were found between visual DT, CRIq total (r = 0.235, *p* = 0.027), and WAIS_voc (r = 0.278, *p* = 0.008) scores.

### 3.2. Regression Models

Only visual creative thinking models were found to be significant. Furthermore, Model 1, Model 3, and Model 4 were significant with age as the only significant predictor in Model 1. Increasing age corresponded to a diminished DT score, whereas higher WAIS_voc scores and CRIq total predicted better visual DT scores (see Table 5).

### 3.3. Mediation Models

**Figure 1:**
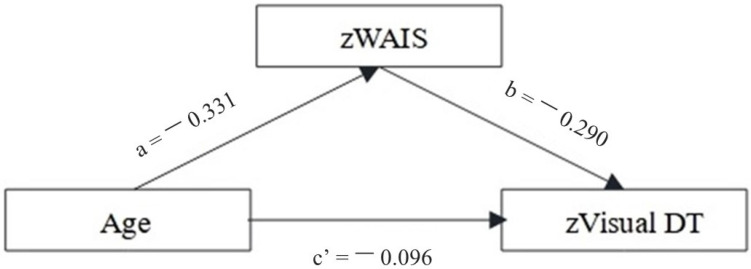
Mediation analysis investigating the role of crystallized intelligence in mediating the relationship between age and visual divergent thinking. Betas were reported for each component (see also Table 6).

Only the WAIS_voc was found to be a significant mediator in the relation between age and visual DT (see Figure 1 and Table 6).

## 4. Discussion

The present study aimed at investigating aging effects on DT abilities and the possible relationships between fluid (i.e., EFs) and crystallized (i.e., WAIS_voc) components of cognition and CR in predicting DT performance in a sample of healthy OAs. We found an aging effect on visual DT performance but a beneficial effect of the crystallized component of cognition together with CR. Moreover, we observed a negative correlation between age and only the originality subscale of verbal DT. 

In line with previous evidence (e.g., [33]), we found that age affected DT performance and visual DT, in particular, in later adulthood. No aging effect was reported instead for most of the verbal DT subscales, possibly indicating that the verbal domain is more stable in the elderly. Indeed, as reported in a previous study, verbal DT begins to decline after middle age and then remains globally stable, whereas visual DT abilities decline in later adulthood [33,54]. If we assume that visual and verbal DT rely on domain-specific processes [55], we should conclude that the verbal domain is less affected than the visual one by cognitive decline. Alternatively, we may also assume that the cognitive load required at different stages of visual and verbal DT, namely, the generation and evaluation of new ideas [36,56], is different in later adulthood and needs different compensation mechanisms. Executive functions/fluid components seem to be more involved in the evaluation stages of DT [31], whereas crystallized ones are more crucial in the generation stage. It follows that, in later adulthood, it could become easier to infer from verbal and autobiographical information (crystallized resources), which are generally stable during the lifespan, than relying on fluid/executive abilities, which are subjected to cognitive decline [6,7,8]. This could implicate differences in the generation and evaluation mechanisms in OAs. Given that verbal crystallized components of cognition are preserved and stable in later adulthood, we may hypothesize that verbal information could be more extended and accessible for the elderly, thus making idea generation less demanding, but evaluation more challenging. Indeed, aging was found to affect only the originality of verbal DT and globally DT performances did not correlate with any of attention and EF measures, indicating the implication of possible alternative cognitive strategies.

Accordingly, during the verbal DT task, individuals are specifically required to generate alternative uses for a given object, and in visual DT, they are required to draw as many figures as possible, without any context information or semantic constraint. This could implicate different cognitive demands and mechanisms involved. From a speculative point of view, assuming that OAs may create privilege for the crystallized component of cognition to complete the DT tasks, we can hypothesize that they rely on it even during visual idea generation. In order to draw figures from two parallel lines, individuals need to imagine the figure first, by accessing their semantic store, and then plan and execute the motor sequence of drawing. These additional cognitive steps implied in visual DT can potentially be more challenging for the elderly, being more cognitively demanding. Indeed, a physiological slowing of responses and a decline in vision together with difficulties in action planning and cognitive control are frequently observed in later adulthood. Consequently, visual DT being possibly more challenging than verbal DT, the amount of CR could play a role in guaranteeing adequate performances even in later adulthood, as shown in the present study, in line with previous evidence [36,37,40,41].

In summary, the positive relationships found between crystallized intelligence and visual DT confirms the pivotal role of semantic and prior knowledge in thinking creatively, allowing connections between weakly related concepts to form new ideas [57,58]. Accordingly, the mediation effect of WAIS_voc in the relation between age and visual DT performance supports the “semanticized” hypothesis of OAs cognition [13,42], where OAs are supposed to rely more on lifelong experiences and knowledge, rather than on fluid and controlled processes, to sustain goal-directed behaviors [13,42]. In this way, our results confirm that, although DT abilities may weaken during aging, if OAs rely on their previous experiences and knowledge as an alternative strategy, they can still perform adequately according to the contingent demands. Moreover, the observed mediative role of crystallized intelligence may underlie a shift toward autobiographical and semantic representations acquired during the lifespan to cope with high cognitive demands, according to the DECHA hypothesis [32].

### Limitations

The study presents some limitations. Firstly, it adopted only one measure for each DT domain (i.e., verbal and visual). Therefore, future studies should apply at least two different measures per domain, to better understand the implication of mechanisms involved in creative cognition in later adulthood. Moreover, we cannot exclude that the individual’s attitude to prefer imagery or verbal strategies [59] in processing information may have influenced the results. Unfortunately, few studies have investigated the influence of cognitive styles on DT, mainly considering visual creativity and finding that a visual cognitive style, rather than a verbal one, can support the generation of original ideas [36]. Further studies should also consider such an individual difference and whether it contributes to sustaining DT in OAs.

## 5. Conclusions

The present study confirmed the strict relationship between DT and cognitive reserve, showing that, even if DT undergoes an age effect, OAs can sustain it by recruiting to a greater extent prior knowledge and crystallized abilities rather than declining control processes, possibly adopting compensatory mechanisms. Such results shed light on the potential role of DT, similar to CR, in counteracting possible cognitive impairments, allowing the recruitment of mostly spared cognitive abilities to functionally perform goal-oriented behaviors and satisfy cognitive demands, even when they are unusual, as it happens in DT tasks.

### Implications

Considering that during aging OAs have to face new situations and difficulties, reporting higher probabilities of experiencing cognitive impairments [60], DT can contribute to coping with everyday life challenges by finding alternative and functional solutions, sustaining cognitive functions [16,17], and it appears crucial to delve into the potential effects that DT can have during later adulthood. In this way, the present findings support the design of effective interventions based on DT for promoting efficient cognitive functioning and sustaining prolonged autonomy. Moreover, these results might have significant practical implications such as the development of specific exercises for OAs aimed at differentially stimulating verbal and visual DT and the relative cognitive strategies. Verbal strategies could be useful in facing the eventual greater cognitive demand required by visual DT performances. On the other hand, stimulating visual DT could also promote more functional visual strategies. Notably, early interventions that specifically aim to expand and make people’s semantic store and networks more flexible could have a beneficial effect on both visual and verbal DT skills, preventing OAs’ decline. More studies are needed to address this issue.

Even if there is a lack of studies that investigated the cognitive mechanisms underlying DT in OAs, it appears important to maintain the focus on the issue, as life expectancy has been prolonged, and the prevention of cognitive impairments and dementia becomes an increasingly crucial issue.

## Figures and Tables

**Table 1:** Mean and standard deviation of sociodemographical, cognitive, and emotional variables of the final sample (*n* = 94).

	Mean	SD
Age	72.27	6.37
Education	12.51	4.55
MMSE	28.4	1.7
CRIq_tot	120.4	18.52
CRIq_e	112.4	14.04
CRIq_w	108.4	19.72
CRIq_la	125.88	22.92
DASS_tot	9.66	7.7
DASS_dep	2.78	3.23
DASS_anx	2.40	2.45
DASS_stress	4.47	3.7

CRIq_tot: Cognitive Reserve Index questionnaire (global score); CRIq_e: Cognitive Reserve Index questionnaire (education score); CRIq_w: Cognitive Reserve Index questionnaire (occupational activities score); CRIq_la: Cognitive Reserve Index questionnaire (leisure activities score); DASS_tot: total score; DASS_dep: Depression Anxiety Stress Scale (depression score); DASS_anx: Depression Anxiety Stress Scale (anxiety score); DASS_stress: Depression Anxiety Stress Scale (stress score).

**Table 2:** Mean and standard deviation of neuropsychological and divergent thinking (visual and verbal DT) outcomes of the final sample (*n* = 94).

Neuropsychological Tests	Mean	SD
TMT_A	43.91	17.95
TMT_B	123.7	56.11
TMT_B-A	79.78	44.2
STROOP_I	24.62	9.28
DIGIT_bw	4.45	1.04
SDMT	40.11	12.94
WAIS_voc	38.54	9.25
DIGIT_fw	5.94	1.07
Story recall	11.32	3.05
**Visual DT**		
Fluency	10.63	5.96
Flexibility	7.78	4.14
Originality	13.46	9.31
Elaboration	11.82	9.23
**Verbal DT**		
Fluency	7.35	3.84
Flexibility	5.22	2.63
Originality	4.24	3.93

DIGIT_bw: Digit Span backward; DIGIT_fw: Digit Span Forward; Story recall: Verbal episodic memory; DT: divergent thinking; SDMT: Symbol Digit Modality Test; Stroop_I: Stroop interference effect measured in time (s); TMT: Trial Making Test time (s), part A (TMT_A), part B (TMT_B), and measure of attentional shifting (B minus A); WAIS_voc: Wechsler Adult Intelligence Scale (vocabulary subtest).

**Table 3:** (**a**) Parametric correlations (cognitive tests) and (**b**) Nonparametric correlations (cognitive tests).

**(a)**		**Age**	**Education**	**ZCRIq_tot**		**ZSTROOP**		**ZSDMT**	**ZWAIS**
education	Correlation coefficient (Pearson)	−0.375 **							
ZCRIq_tot	Correlation coefficient (Pearson)	−0.220 *	0.610 **						
ZSTROOP	Correlation coefficient (Pearson)	0.385 **	−0.240 *	−0.153					
ZSDMT	Correlation coefficient (Pearson)	−0.510 **	0.517 **	0.308 **		−0.351 **			
ZWAIS	Correlation coefficient (Pearson)	−0.314 **	0.466 **	0.303 **		−0.252 *		0.248 *	
**(b)**		**Age**	**Education**	**ZTMT_B**	**ZTMT_A**	**ZTMT_B-A**	**ZDIGIT_fw**	**ZDIGIT_bw**	**Z_Story** **recall**
education	Correlation coefficient (Spearman)	−0.388 **							
ZTMT_B	Correlation coefficient (Spearman)	0.461 **	−0.453 **						
ZTMT_A	Correlation coefficient (Spearman)	0.456 **	−0.299 **	0.681 **					
ZTMT_B-A	Correlation coefficient (Spearman)	0.398 **	−0.472 **	0.967 **	0.497 **				
ZDIGIT_fw	Correlation coefficient (Spearman)	−0.255 *	0.315 **	−0.341 **	−0.265 *	−0.330 **			
ZDIGIT_bw	Correlation coefficient (Spearman)	−0.326 **	0.298 **	−0.339 **	−0.278 **	−0.345 **	0.379 **		
Z_Story recall	Correlation coefficient (Spearman)	−0.114	0.127	−0.027	−0.042	−0.012	0.000	0.335 **	

(**a**) CRIq_tot: Cognitive Reserve Index questionnaire (global score); SDMT: Symbol Digit Modality Test; Stroop_I: Stroop interference effect measured in time (seconds); WAIS_voc: Wechsler Adult Intelligence Scale (vocabulary subtest). The prefix Z refers to z scores. ** Significance two tails: *p* < 0.005; * Significance two tails: *p* < 0.05. (**b**) DIGIT_bw: Digit Span backward; DIGIT_fw: Digit Span Forward; Story recall: Verbal episodic memory; TMT: Trial Making Test time (seconds), part A (TMT_A), part B (TMT_B), and measure of attentional shifting (B minus A). The prefix Z refers to Z scores. ** Significance two tails: *p* < 0.005; * Significance two tails: *p* < 0.05.

**Table 4:** Nonparametric correlations (visual and verbal divergent thinking subscales).

		Age	Education	zVISUAL_flu	ZVISUAL_flex	ZVISUAL_or	ZVISUAL_el	ZVERBAL_flu	ZVERBAL_flex	ZVERBAL_or
education	Correlation coefficient (rho Spearman)	−0.388 **								
zVISUAL_flu	Correlation coefficient (rho Spearman)	−0.379 **	0.232 *							
ZVISUAL_flex	Correlation coefficient (rho Spearman)	−0.358 **	0.208 *	0.892 **						
ZVISUAL_or	Correlation coefficient (rho Spearman)	−0.285 **	0.078	0.870 **	0.829 **					
ZVISUAL_el	Correlation coefficient (rho Spearman)	−0.313 **	0.132	0.525 **	0.458 **	0.513 **				
ZVERBAL_flu	Correlation coefficient (rho Spearman)	−0.101	0.047	0.254 *	0.230 *	0.293 **	0.269 **			
ZVERBAL_flex	Correlation coefficient (rho Spearman)	−0.132	0.032	0.302 **	0.242 *	0.312 **	0.202	0.871 **		
ZVERBAL_or	Correlation coefficient (rho Spearman)	−0.191+	0.022	0.361 **	0.335 **	0.348 **	0.134	0.781 **	0.782 **	

el: elaboration; flex: flexibility; flu: fluency; or: originality. The prefix Z refers to z scores. ** Significance two tails: *p* < 0.005; * Significance two tails: *p* < 0.05; + *p* = 0.06.

**Table 5:** Hierarchical regressions with visual DT as dependent variable.

Model	R	R2	Adjusted R2	Std. Error	R2 Change	F Change	Sign. F Change
1	0.445	0.198	0.170	0.78617	0.198	7.161	0.000
2	0.450	0.203	0.146	0.79771	0.005	0.167	0.918
3	0.516	0.266	0.204	0.77005	0.063	7.144	0.009
4	0.556	0.309	0.241	0.75178	0.043	5.082	0.027
**Model coefficients**							
**Model**	**Variables**	**β**	**t**	**Significance (*p* value)**			
1	Age	−0.418	−4.019	<0.001			
1	Education	0.072	0.689	0.493			
1	Gender	0.039	0.402	0.689			
2	Age	−0.384	−3.223	0.002			
2	Education	0.036	0.298	0.767			
2	Gender	0.035	0.347	0.729			
2	Executive composite score	−0.047	−0.390	0.697			
2	ZSDMT	0.030	0.226	0.822			
2	ZDIGIT_bw	0.037	0.333	0.740			
3	Age	−0.332	−2.848	0.006			
3	Educaton	−0.085	−0.684	0.496			
3	Gender	0.021	0.217	0.829			
3	Executive composite score	0.006	0.054	0.957			
3	ZSDMT	0.058	0.449	0.655			
3	ZDIGIT_bw	0.063	0.586	0.560			
3	ZWAIS_voc	0.296	2.673	0.009			
4	Age	−0.335	−2.940	0.004			
4	Education	−0.245	−1.740	0.086			
4	Gender	0.008	0.083	0.934			
4	Executive composite score	0.024	0.203	0.839			
4	ZSDMT	0.060	0.476	0.636			
4	ZDIGIT_bw	0.072	0.683	0.496			
4	ZWAIS_voc	0.296	2.732	0.008			
4	ZCRIq_tot	0.263	2.254	0.027			

CRIq_tot: Cognitive Reserve Index questionnaire (global score); DIGIT_bw: Digit Span backward; SDMT: Symbol Digit Modality Test; WAIS_voc: Wechsler Adult Intelligence Scale (vocabulary subtest).

**Table 6:** Summary of mediation analysis considering WAIS_voc as a mediator in the relation between age and visual DT. Indirect and direct effects are summarized below.

Type		Effect	Estimate	SE	95% C.I. (a)		β	z	Significance (*p* Value)
					Lower	Upper			
Indirect	c’	age ⇒ zWAIS ⇒ zvisuaDT	−0.013	0.005	−0.022	−0.003	−0.096	−2.500	0.013
Component	a	age ⇒ zWAIS	−0.049	0.015	−0.078	−0.020	−0.331	−3.290	0.001
	b	zWAIS ⇒ zvisualDT	0.257	0.070	0.119	0.395	0.290	3.650	<0.001
Direct	c	age ⇒ zvisualDT	−0.041	0.012	−0.065	−0.017	−0.315	−3.410	<0.001
Total	c + (a × b)	age ⇒ zvisualDT	−0.054	0.013	−0.079	−0.029	−0.411	−4.250	<0.001

DT: divergent thinking; WAIS_voc: Wechsler Adult Intelligence Scale (vocabulary subtest).

## Data Availability

The dataset will be made available upon request.
